# A novel electrochemical aptasensor for fumonisin B_1_ determination using DNA and exonuclease-I as signal amplification strategy

**DOI:** 10.1186/s13065-019-0646-z

**Published:** 2019-11-09

**Authors:** Min Wei, Fei Zhao, Shuo Feng, Huali Jin

**Affiliations:** College of Food Science and Technology, Henan Key Laboratory of Cereal and Oil Food Safety Inspection and Control, Henan University of Technology, Zhengzhou, 450001 People’s Republic of China

**Keywords:** Electrochemical aptasensor, Fumonisin B_1_, Exonuclease-I, G-rich DNA, Methylene blue

## Abstract

In this work, using DNA and exonuclease-I (Exo-I) as signal amplification strategy, a novel and facile electrochemical aptasensor was constructed for fumonisin B_1_ (FB_1_) detection. The G-rich complementary DNA (cDNA) was immobilized onto the electrode surface. Then, aptamer of FB_1_ was hybridized with cDNA to form double-stranded DNA. In the absence of FB_1_, double-stranded DNA and G-rich cDNA on the electrode surface promoted effectively methylene blue (MB) enrichment and amplified the initial electrochemical response. In the presence of FB_1_, the combination of aptamer and FB_1_ led to the release of aptamer from the electrode surface and the expose of 3′ end of single-stranded cDNA. When Exo-I was added onto the electrode surface, the single-stranded cDNA was degraded in the 3′–5′ direction. The decrease of double-stranded DNA and G-rich cDNA resulted in the less access of MB to the electrode surface, which decreased the electrochemical signal. The experimental conditions including incubation time of FB_1_, the amount of Exo-I and incubation time of Exo-I were optimized. Under the optimal conditions, the linear relationship between the change of peak current and the logarithmic concentration of FB_1_ was observed in the range of 1.0 × 10^−3^–1000 ng mL^−1^ with a low limit of detection of 0.15 pg mL^−1^. The experimental results showed that the prepared aptasensor had acceptable specificity, reproducibility, repeatability and stability. Therefore, this proposed aptasensor has a potential application in the food safety detection.

## Introduction

As the metabolic product of Fusarium moniliforme Sheld, fumonisin B_1_ (FB_1_) is a kind of the most toxic and prevalent fumonisins [1]. FB_1_ can contaminate various food and feedstuff such as corn, wheat, rice, peanut, beer, and animal feed. A large number of studies have reported that FB_1_ can cause serious diseases such as horse white matter softening, nephrotoxicity, hepatotoxicity and liver cancer [2, 3]. Therefore, it is necessary to monitor FB_1_ for food safety and human health.

Among the various methods for FB_1_ detection [4–7], the electrochemical aptasensor has attracted widespread attention due to their low cost, simple operation, high selectivity and affinity, chemical stability, and easy storage [8, 9]. Recently, with the advantages including effective amplification strategy, easy design, simple operation and rapid reaction, the nuclease-based electrochemical aptasensor has become research focus [10, 11]. Among the different nucleases, exonuclease I (Exo-I) has attracted increasing attention, owing to its structure-sensitive digestion for the single-stranded DNA in the direction of 3′ to 5′, low cost, good specificity and buffer compatibility [12–14]. As a kind of electrochemical signal probe, methylene blue (MB) can highly interact with G-rich single-stranded DNA and double-stranded DNA, and is therefore suitable for the application in electrochemical aptasensor [15, 16].

Herein, based on MB, Exo-I, aptamer of FB_1_ (Apt) and G-rich cDNA, a novel signal-off sensor was firstly designed for the electrochemical detection of FB_1_. The existing double-stranded DNA on the electrode surface, came from the hybridization of Apt and G-rich cDNA, enriched abundant MB and amplified the initial electrochemical response. In the presence of FB_1_, the formation of Apt-FB_1_ made aptamer release from the electrode surface. Then, the effect of Exo-I on G-rich cDNA of the electrode surface resulted in the less access of MB, which further decreased the electrochemical signal and amplified ΔI. The change of MB electrochemical signal can be applied for FB_1_ detection.

In virtue of the favorable combination of MB with double-stranded DNA and G-rich cDNA, and the advantages of Exo-I including easy design, simple operation, high amplification efficiency and excellent selectivity, the proposed signal amplification strategies can save the tedious preparation process and is beneficial to the experimental stability.

## Experimental

### Materials and chemicals

The used oligonucleotides were provided by Sangon Biological Engineering Technology & Services Co. Ltd. (Shanghai, China), and their sequences were as follows: cDNA: 5′-SH-GAG GGG TGG GCG GGA GGG AGA TTG CAC GGA CTA TCT AAT TGA ATA AGC-3′. Apt: 5′-ATA CCA GCT TAT TCA ATT AAT CGC ATT ACC TTA TAC CAG CTT ATT CAA TTA CGT CTG CAC ATA CCA GCT TAT TCA AGT AGA TAG TAA GTG CAA TCT-3′. FB_1_ and Exo-I were purchased from Acros and TaKaRa, respectively. 0.05 M of pH 7.4 Tris–HCl buffer (containing 0.05 M Tris, 0.2 M NaCl and 0.001 M EDTA) was used.

### Apparatus

The CHI 660E Electrochemical Workstation was used for the electrochemical experiments (Shanghai Chenhua Instrument Corporation, China). The gold electrode (AuE) was used as working electrode. Differential pulse voltammetry (DPV) and electrochemical impedance spectroscopy (EIS) were used for the electrochemical measure.

### Fabrication and mechanism of the aptasensor

**Fig. 1:**
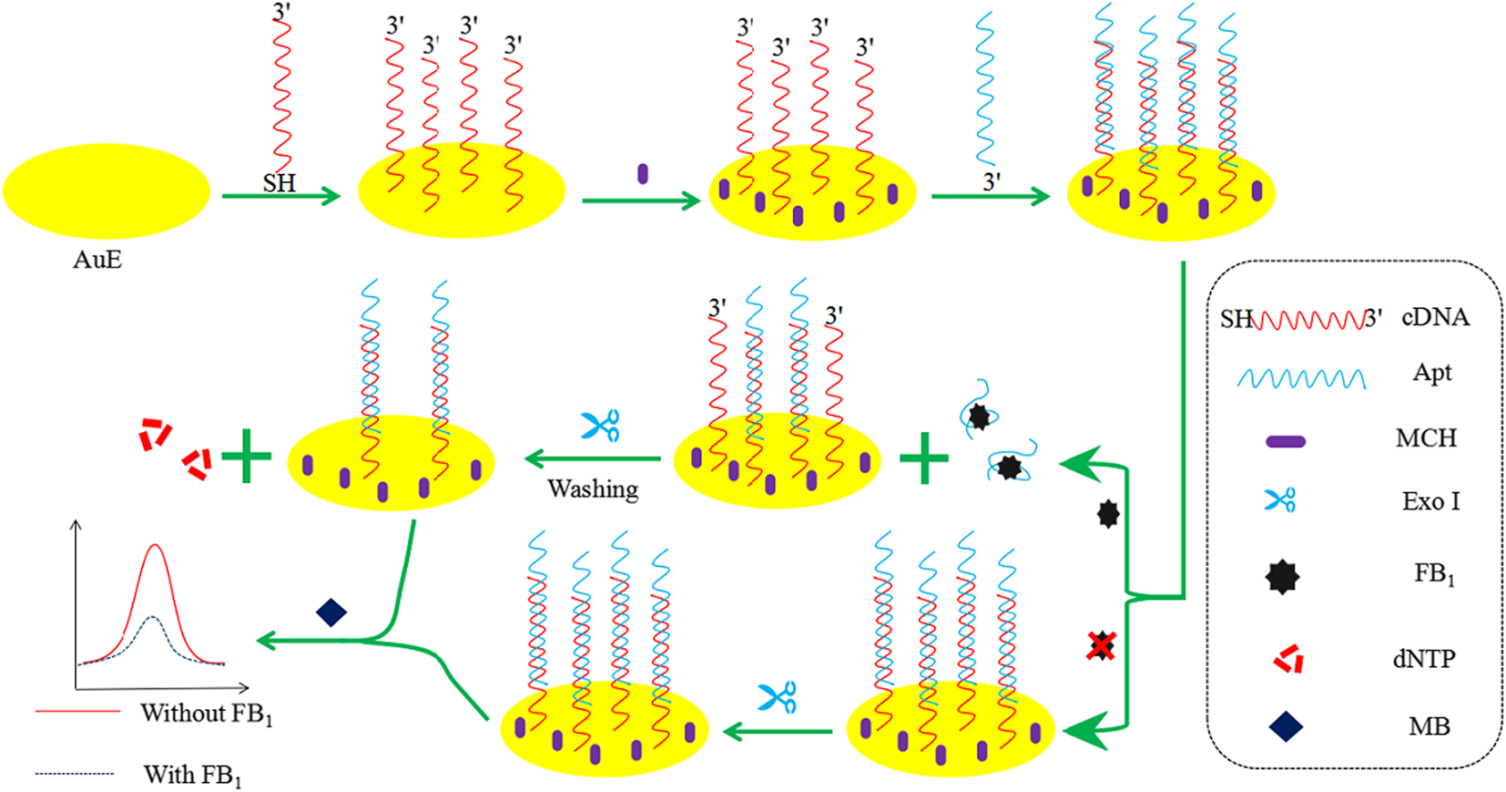
The fabrication and mechanism of the aptasensor

The fabrication and mechanism of the aptasensor were shown in Fig. 1. 5 μL of 1 μM SH-cDNA was dropped on the AuE surface for immobilization at 37 °C. Then, the AuE was washed by Tris–HCl buffer to remove the unbound cDNA. After that, 5 μL of 6-mercapto-1-hexanol (MCH) was dropped to block the untreated sites. Next, 5 µL of 1 μM Apt was hybridized with cDNA for 2 h at 37 °C to obtain the aptasensor Apt/cDNA/AuE.

When FB_1_ was absent, cDNA and Apt could not be degraded by Exo-I because that the 3′ end of both cDNA and Apt were protected by the formation of double-stranded DNA. MB could intercalate into G-rich cDNA and double-stranded DNA, and produce a strong current signal. When FB_1_ was present, the complex of Apt and FB_1_ was formed and released from the surface of the electrode, leading to the expose of 3′ end of single-stranded cDNA on the electrode surface. When Exo-I was added onto the electrode surface, the single-stranded cDNA was degraded in the 3′-5′ direction. The decrease of double-stranded DNA and G-rich cDNA resulted in the less access of MB to the electrode surface and the decrease of the electrochemical signal. The change of MB electrochemical signal can be applied for FB_1_ detection.

## Results and discussion

### Electrochemical characterization of the prepared aptasensor

**Fig. 2:**
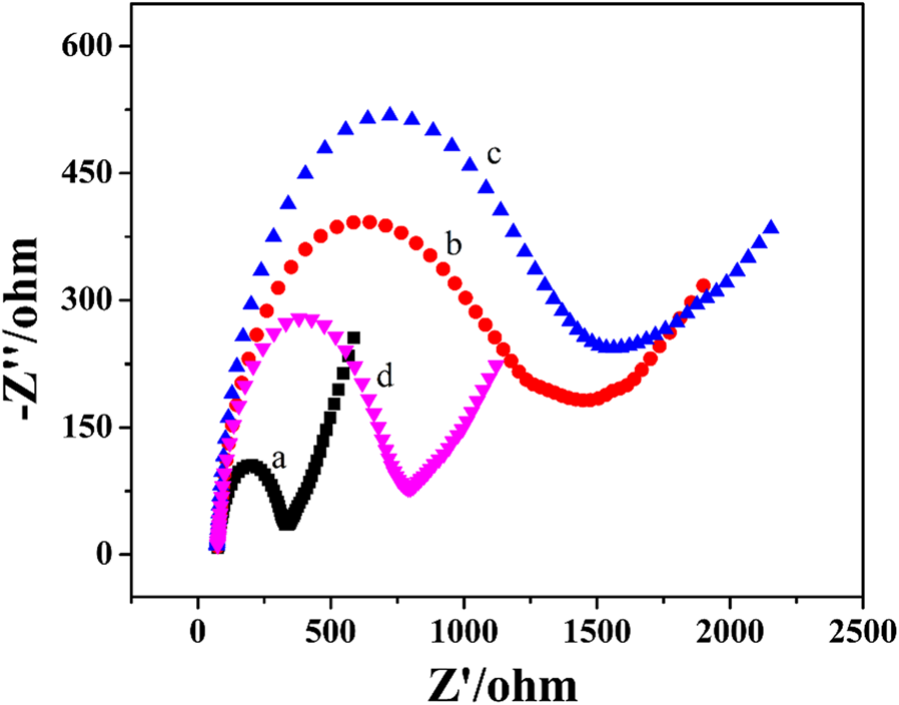
EIS of 10 mM [Fe(CN)_6_]^3−/4−^ containing 0.1 M KCl on the AuE (a), the cDNA/AuE (b), and the Apt/cDNA/AuE before (c) and after (d) addition of FB_1_ and Exo-I

Figure 2 showed the EIS characterization for the aptasensor fabrication. The charge transfer resistance (R_ct_) increased from 251.3 Ω of the bare AuE (a) to 1219 Ω of the cDNA/AuE (b), indicating that the cDNA was immobilized to the electrode surface. For the Apt/cDNA/AuE (c), the R_ct_ was increased to 1381 Ω, indicating that Apt hybridized successfully with cDNA on the electrode surface. After the Apt/cDNA/AuE was incubated by 1 μg mL^−1^ FB_1_ and Exo-I, respectively, the R_ct_ (d) was decreased to 836 Ω. This was because that Apt was specifically combined with FB_1_ and released from the electrode, and cDNA was digested by Exo-I due to the expose of its 3′ end, resulting in the less negative charge on the electrode surface.

### The detection of FB_1_ on Apt/cDNA/AuE sensor

**Fig. 3:**
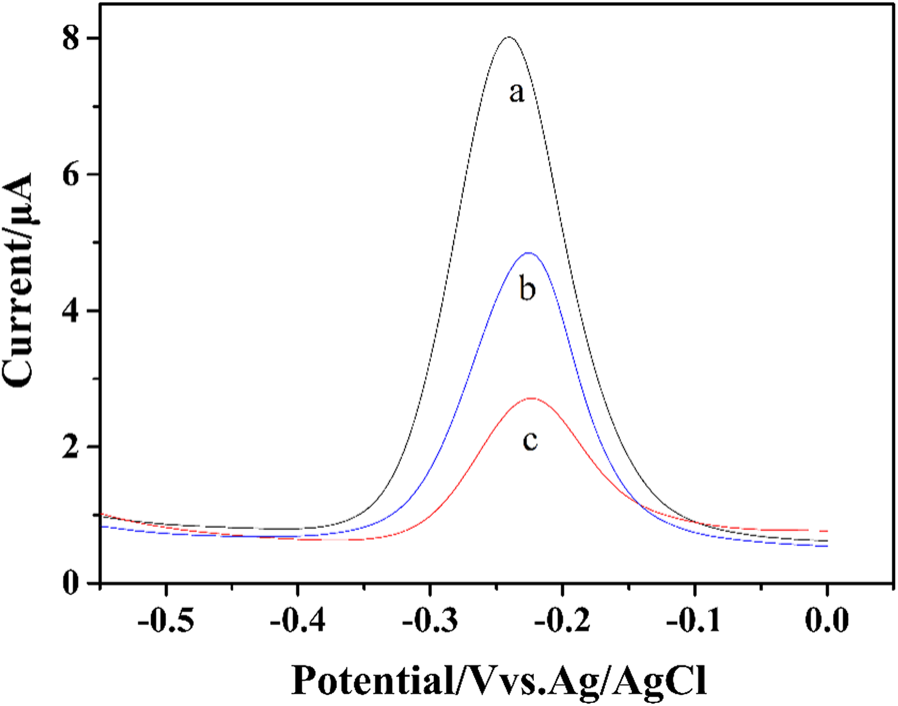
The DPV results of MB on the Exo-I/Apt/cDNA/AuE (a), FB1/Apt/cDNA/AuE (b) and Exo-I/FB_1_/Apt/cDNA/AuE (c) in Tris–HCl buffer

Figure 3 showed the DPV results of MB on the Exo-I/Apt/cDNA/AuE (a), FB_1_/Apt/cDNA/AuE (b) and Exo-I/FB_1_/Apt/cDNA/AuE (c) in Tris–HCl buffer. In the absence of FB_1_, the Exo-I/Apt/cDNA/AuE showed an initial peak current of 7.29 μA (a). With the addition of 1 μg·mL^−1^ FB_1_, the peak current of FB_1_/Apt/cDNA/AuE (b) decreased to 4.02 μA. This is because that in the presence of FB_1_, the formation of Apt-FB_1_ composite made Apt release from double-stranded DNA on the electrode surface, resulting in that the amounts of MB intercalated into the double-stranded DNA were decreased. After the addition of Exo-I, the DPV value of Exo-I/FB_1_/Apt/cDNA/AuE (c) further decreased to 2.41 μA, indicating that Exo-I could digest the single-stranded cDNA on the electrode surface and achieve the signal amplification.

### Optimization of the aptasensor

**Fig. 4:**
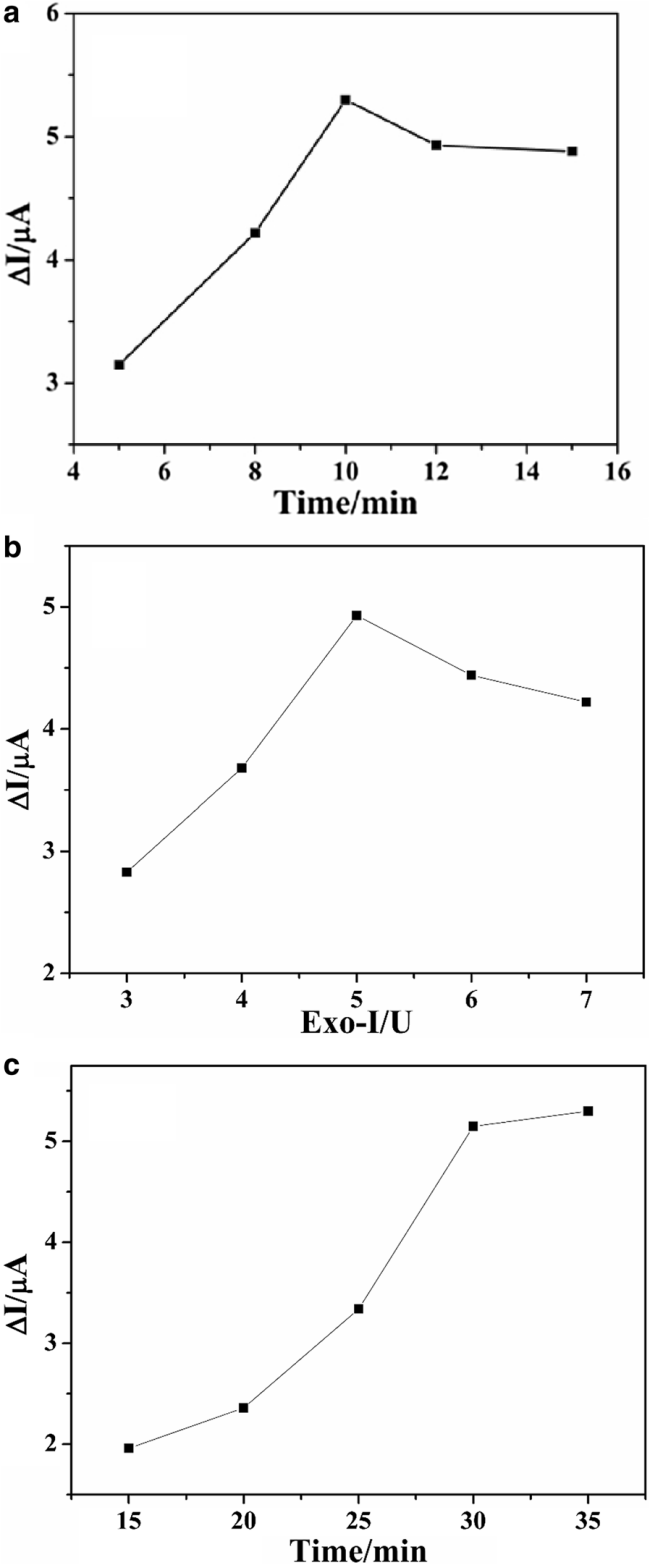
The effect of FB_1_ incubation time (**a**), Exo-I amount (**b**) and Exo-I incubation time (**c**) on the electrochemical signal

Figure 4 showed the effect of FB_1_ incubation time (A), Exo-I amount (B) and Exo-I incubation time (C) on the electrochemical signal. As shown in Fig. 4a, it can be seen that ΔI increased with the increasing of FB_1_ incubation time and reached the maximum of 5.3 μA at 10 min. Therefore, 10 min was selected as the optimal FB_1_ incubation time. As can be seen from Fig. 4b, ΔI increased with increasing of Exo-I amount and reached the maximum at 5 U, then decreased when the amount was further increased. This may due to that the limit of active surface area on the fabricated electrode led to the inefficiency of redundant Exo-I. So, 5 U of Exo-I was used for the subsequent experiments. As shown in Fig. 4c, the ΔI increased quickly with increasing the incubation time in the first 30 min, then changed slightly when the incubation time was more than 30 min. Therefore, 30 min was used as the optimal Exo-I incubation time.

### Analytical performance of the designed aptasensor

**Fig. 5:**
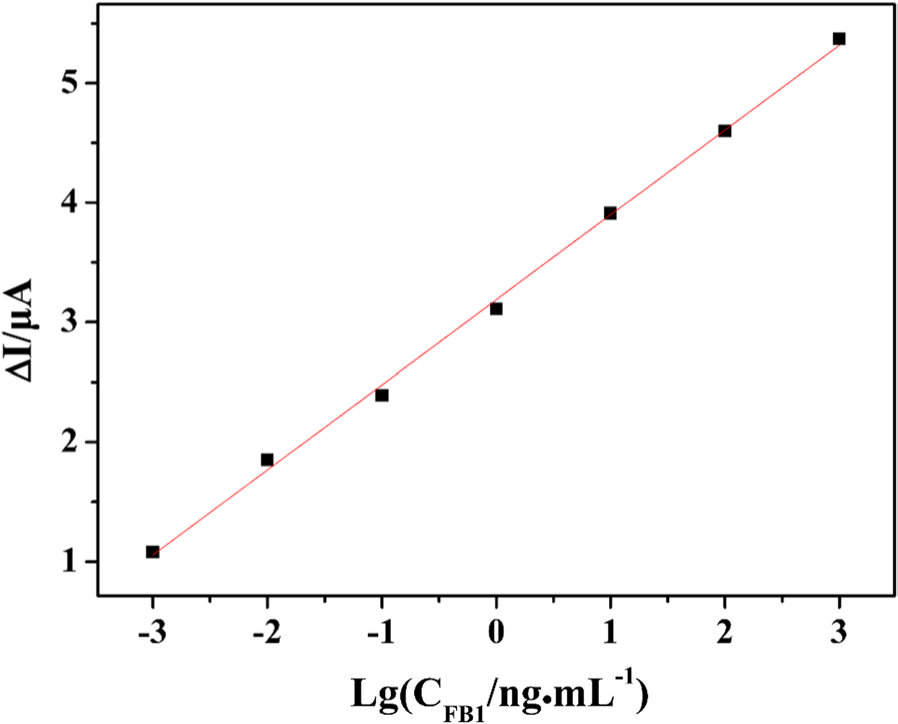
The linear relationship between ∆I and Lg[C_FB1_] with FB_1_ concentration in the range of 1 × 10^−3^ ~ 1000 ng mL^−1^

Figure 5 showed the calibration plot of the fabricated aptasensor for FB_1_ detection. With the concentration range of 1 × 10^−3^~1000 ng·mL^−1^, a linear relationship between ∆I and Lg [C_FB1_] was observed, and the linear regression equation was ∆I = 0.71036 Lg[C_FB1_] + 3.18714 (R^2^ = 0.998). The limit of detection (LOD) was calculated to be 0.15 pg·mL^−1^ at a signal-to-ratio of 3. Compared to the previous reports, the designed aptasensor obtained a wider linear range and lower LOD, and the result was shown in Table 1.

**Table 1:** Comparison with other reported methods for FB_1_ detection

Method	Amplification strategy	Linearity, (ng mL^−1^)	LOD, (ng mL^−1^)	Refs.
Chemiluminescence and enzyme-linked immunosorbent	ECL-ELISA based on anti-FB_1_ IgG and HRP	0.14–0.9	0.09	[17]
Chemiluminescence	Charge-coupled device	2.5–500	2.5	[18]
Fluorescence	Anti apt/Apt-NH_2_/TiO_2_-PSi	0.001–10	0.21 × 10^−3^	[5]
Fluorescence resonance energy transfer	AuNPs-MB-UCNPs	0.01–100	0.01	[19]
Electrochemiluminescence	MIP/Ru@SiO_2_/CS/AuNPs/GCE	1 × 10^−3^ ~ 100	0.35 × 10^−3^	[6]
Electrochemical immunosensor	AP-anti-antibody/anti-FB_1_/FB_1_-BSA-SWNTs/CS/GCE	0.01–1000	2 × 10^−3^	[20]
Electrochemical immunosensor	Ab-AuNPs-PPy/ErGO-SPE	200–4500	4.2	[21]
Electrochemical magneto immunosensor	FB1-HRP/Ab-FB_1_/MB&protein G/CSPE	0.73–11.2	0.33	[22]
Electrochemical aptasensor	Apt-AuNPs-SPCE	1 × 10^−2^~50	3.4 × 10^−3^	[7]
Electrochemical aptasensor	GS-TH/S2/S1/Au/GCE	1 × 10^−3^~1000	1 × 10^−3^	[23]
Electrochemical aptasensor	Exo-I/Apt/cDNA/AuE	1 × 10^−3^~1000	0.15 × 10^−3^	This work

### Specificity, reproducibility, repeatability and stability

**Fig. 6:**
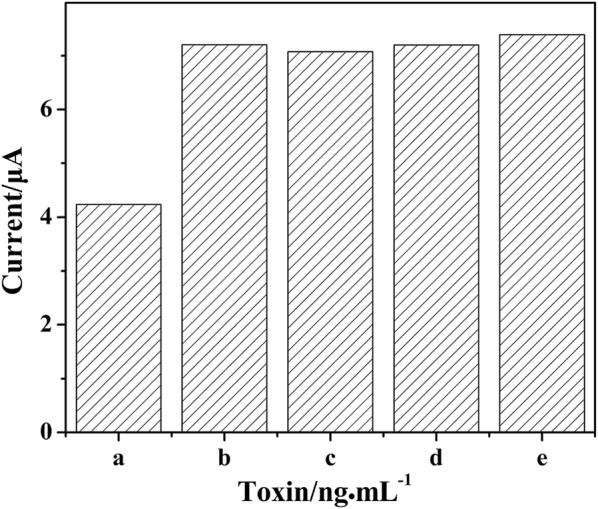
The peak current of aptasensor incubated in different toxins with the same concentration of 1 ng mL^−1^. a FB_1_, b OTA, c ZEA, d AFB_1_, e Blank

The specificity of the aptasensor to ochratoxin A (OTA), zearalenone (ZEA) and aflatoxin B_1_ (AFB_1_) was studied, and the results were shown in Fig. 6. Only when the prepared aptasensor was incubated in FB_1_, the peak current decreased significantly, indicating that the designed aptasensor had good specificity and could meet the experimental requirements.

Under the optimized conditions, the reproducibility and the repeatability of the fabricated aptasensor was respectively evaluated with inter-assay and intra-assay. Under the same experimental conditions, five fabricated aptasensors were tested by monitoring the peak current of MB with 1 μg mL^−1^ FB_1_ on the FB_1_/Apt/cDNA/AuE, and a relative standard deviation (RSD) of 5.72% was calculated, implying that the fabricated sensor had satisfactory reproducibility. The one aptasensor was investigated by monitoring the peak current of MB in the presence of 1 μg mL^−1^ FB_1_ for five replicate determinations under the same conditions, and RSD of 5.38% was calculated, implying that the fabricated aptasensor had acceptable repeatability.

For the study on stability of the fabricated aptasensor, the peak current of MB on the three Exo-I/Apt/cDNA/AuE was detected, and the average peak current is 7.21 μA. Then the fabricated aptasensors were stored at 4 °C. After a 35-day storage period, the average peak current of MB on the Exo-I/Apt/cDNA/AuE was 6.14 μA, and the aptasensor retained 85.2% of its initial current response, indicating the acceptable stability.

### Analysis of FB_1_ in food samples

The accuracy of the fabricated aptasensor was evaluated by studying the recovery of FB_1_ in beer samples and corn samples, and the results were shown in Table 2. Beer samples were filtrated through a 0.45 µm membrane, and used for subsequent tests by spiking different concentrations of FB_1_. Non-contaminated corn samples were finely milled to obtain corn powder, and 0.5 g of the corn powder was extracted with methanol–water (60:40, v/v. 5 mL) using an orbital shaker for 30 min. After centrifugation for 15 min, the extract was used for analysis by spiking different concentrations of FB_1_. By addition of 100 ng mL^−1^, 1 ng mL^−1^ and 1 × 10^−2^ ng mL^−1^ of FB_1_, for the beer samples, the average recoveries were 88.5%, 96.1% and 98.6%, respectively. For the corn samples, the average recoveries were 91.4%, 87.3% and 106.8%, respectively. These results indicated that the fabricated aptasensor can be applied in FB_1_ detection of the food samples.

**Table 2:** Recovery of FB_1_ in food samples

Sample	Added (ng mL^−1^)	Average found (ng mL^−1^)	Average recovery (%)	RSD (%), n = 3
Beer	100	88.5	88.5	1.75
	1	0.961	96.1	2.25
	1 × 10^−2^	0.986 × 10^−2^	98.6	5.65
Corn	100	91.4	91.4	4.76
	1	0.873	87.3	7.79
	1 × 10^−2^	1.068 × 10^−2^	106.8	6.34

## Conclusion

In summary, on the basis of DNA and Exo-I as signal amplification strategy, a novel and facile signal-off aptasensor was developed for FB_1_ detection. Utilizing the favorable combination of MB with double-stranded DNA and G-rich cDNA, the specific DNA was designed to enrich abundant MB for initial signal amplification. On the other hand, with the advantages of easy design, simple operation, high amplification efficiency and excellent selectivity, Exo-I was used to design a novel signal-off aptasensor for amplifying the ΔI. These two signal amplification strategies can avoid the complicated nanomaterial preparation and instability. As a result, this proposed aptasensor showed the favorable performance with simple preparation, good selectivity, reproducibility, repeatability, stability as well as a wider linear range with lower LOD, providing a promising potential for application in food safety detection.

## Data Availability

All data generated or analyzed during this study are included in this published article. We have presented all data in the form of tables and figures.

## Abbreviations

DNA: deoxyribonucleic acid
Exo-I: exonuclease-I
FB_1_: fumonisin B_1_
cDNA: complementary DNA
MB: methylene blue
Apt: aptamer of FB_1_
DPV: differential pulse voltammetry
EIS: electrochemical impedance spectroscopy
MCH: 6-mercapto-1-hexanol
AuE: the gold electrode
R_ct_: the charge transfer resistance
∆I: the difference of peak current
LOD: limit of detection
OTA: ochratoxin A
ZEA: zearalenone
AFB_1_: aflatoxin B_1_
RSD: relative standard deviation
